# Unveiling antimicrobial stewardship competence among Italian nurses: results from a nationwide survey

**DOI:** 10.1186/s13756-025-01531-8

**Published:** 2025-02-23

**Authors:** Matteo Danielis, Tania Buttiron Webber, Chiara Barchielli, Maria Mongardi, Domenico Regano

**Affiliations:** 1 Laboratory of Studies & Evidence Based Nursing, Department of Cardiac, Thoracic, Vascular Sciences and Public Health, University of Padova, Via Loredan 18, Padova, 35131 Italy; 2 ANIPIO, Società Scientifica Nazionale degli Infermieri Specialisti del Rischio Infettivo - National Association of Nurses for the Prevention of Hospital Infections, Bologna, Italy; 3 Medical Oncology, Galliera Hospital, Mura delle Cappuccine 14, Genoa, 16128 Italy; 4 Management and Health Laboratory, Institute of Management, Sant’Anna School of Advanced Studies, Pisa, 56127 Italy; 5 Department of Medicine and Surgery, University of Parma, Via Gramsci 14, Parma, 43126 Italy; 6 Hospital Hygiene and Prevention Unit, IRCCS Azienda Ospedaliero-Universitaria di Bologna, Via Albertoni 15, Bologna, 40138 Italy

**Keywords:** Antimicrobial stewardship, Nursing competence, Italian healthcare, Nationwide survey, Infection control

## Abstract

**Background:**

The development of nursing competencies in antimicrobial stewardship (AMS) is influenced by a two-dimensional model, encompassing both internal and environmental factors. In the context of Italian clinical nursing, this study aims to assess and measure these aspects.

**Methods:**

Employing a cross-sectional online survey design, nurses from various clinical specialties in Italy were involved. The questionnaire assessed individual variables, knowledge, attitudes, practices, as well as structural and process-related variables influencing AMS. Statistical analyses were performed, encompassing descriptive statistics, Pearson correlation, and multiple linear regression.

**Results:**

A total of 1,651 nurses aswered the survey, with a participation rate of 50.8%. The participant demographic revealed that 77% were female, and they had an average of 15 years of experience. Workplace and regional factors were found to significantly impact both AMS practices and attitudes. Surgical nurses reported higher practices scores (β = 0.467, *P* <.01), while critical care nurses scored lower (β= -0.398, *P* <.01). Regarding perceptions of structure, workplace characteristics significantly influenced nurses’ scores, indicating lower perceptions among surgical nurses compared to their medical counterparts (β= -0.315, *P* <.01).

**Conclusions:**

The study reveals the intricate interplay between internal and external factors that impact nurses’ AMS competence. This underscores the urgent need for targeted interventions and education initiatives to enhance nurses’ AMS competencies. Specifically, addressing variations in settings and nurses’ behaviours becomes imperative for achieving improved patient outcomes.

## Background

A comprehensive assessment of the global burden of antimicrobial resistance (AMR), based on estimates for 204 countries and territories, projected 4.71 million deaths associated with bacterial AMR in 2021. Out of these, 1.14 million deaths were directly attributable to drug resistance [1]. Given the significant healthcare challenge posed by bacterial AMR, it is imperative to identify effective methods to mitigate its impact. Moreover, responsible utilization of antimicrobials necessitates active participation from healthcare professionals (HCPs), underscoring their pivotal role in addressing this pressing issue [2]. The nurse’s role in antimicrobial stewardship (AMS) aimed at the responsible use of antimicrobials to preserve their future effectiveness, has garnered widespread recognition [3].

The Centers for Disease Control and Prevention (CDC) and the American Nurses Association (ANA) have delineated six discrete functions for nurses within the framework of AMS [4]. These roles encompass the following: (a) collect high-quality culture specimens before administering antimicrobials; (b) guide antimicrobial selection and discontinuation for colonized patients based on culture results; (c) collaborate to ensure timely initiation of antimicrobial therapy for bacterial infections, including sepsis; (d) integrate antimicrobial therapy into quality improvement, ensuring timely treatment for sepsis based on culture results; (e) participate in discussions on antimicrobial therapy, considering transitions and de-escalation based on ongoing patient assessment; and (f) educate patients and families on the patient’s penicillin allergy history.

Most of the available quantitative studies available on this topic have focused on nursing knowledge about AMR, AMS, or stewardship-related activities. Meanwhile, findings from qualitative studies have predominantly explored nurses’ attitudes toward AMS [5].

Furthermore, many studies have reported results on the healthcare workforce as a whole [6], highlighting a lack of literature specifically focusing on nurses [7]. In addition, little is known about the factors that promote or hinder nurse involvement in AMS activities. A descriptive online survey, encompassing 343 nurses across three American hospitals, revealed that 52% exhibited no familiarity with the term AMS, while 27% acknowledged having heard of the term but lacked comprehension of its meaning [8]. Remarkably, individuals exhibiting a high level of knowledge in AMS were distinguished by both familiarity with the term and a recognition of its significance. This underscores the correlation between awareness, comprehension, and the perceived importance of an AMS program within their respective settings. A parallel investigation was carried out across 11 hospitals in Pakistan, primarily addressing the authors’ concern regarding the prevailing lack of participation among nurses in AMS programs [9]. Specifically, less than one-third of the study cohort was currently involved in various facets of AMS. Additionally, a considerable proportion, approximately 50.1%, had never taken proactive measures in addressing problematic prescriptions, and 56.3% refrained from commenting on medical orders. These findings indicate a significant deficit in active involvement in critical aspects of AMS practices among the surveyed nursing population. It is evident that a heavy workload poses a substantial obstacle for nurses to actively participate in AMS hospital programs [9, 10].

Nation-specific surveys have been conducted to assess AMS competencies among nurses. As an example, a recent explanatory sequential mixed-methods study evaluated Chinese bedside nurses’ knowledge, attitudes, and practices regarding AMS. Nurses scored 75% in knowledge, 82.8% in attitude, and 84.1% in practice domains. While nurses demonstrate positive attitudes and practices, addressing the existing knowledge gap remains crucial for improving antimicrobial stewardship in nursing [11]. Nursing competence in AMS seems to be governed by a two-dimensional model. On one axis are internal factors encompassing knowledge, attitudes, and practices [12]. On the other axis are external factors, situated at the environmental level, including structures and processes. This dual framework underscores the multifaceted nature of the influences shaping nursing proficiency in AMS, emphasizing the interplay between individual attributes and broader contextual elements in healthcare settings [12]. A recent cross-sectional, multicenter survey was conducted in Italy, targeting nurses, nursing students, and other HCPs [13]. The study utilized an anonymous online questionnaire that focused on AMR. The survey garnered responses from 848 participants, of which a significant majority (61.9%) were students. It’s important to note that the authors acknowledged a limitation of the study, stating that the sample was not representative of the entire country.

Given the absence of prior studies examining the specified dimensions of AMS competence among nurses in Italy, this study aims to bridge this knowledge gap on a large national scale, with a particular focus on clinical nurses. The primary aim of this study was to assess the knowledge, attitudes and practices related to antimicrobial resistance and stewardship among Italian clinical nurses. The secondary aim was to identify external factors, situated at the environmental level in terms of structures and processes, that influence nurses’ participation in AMS programs.

## Methods

### Study design

The study is a cross-sectional survey design. Results are reported according to the Strengthening the Reporting of Observational Studies in Epidemiology (STROBE) checklist for cross-sectional studies [14].

### Setting and sample

The recruitment strategy employed a nationwide, purposive sampling approach [15]. All clinical public settings within the national health system (e.g., medical and surgical areas) were included in the study. The Italian Society for Professionals in Infection Control (ANIPIO) played a crucial role in this process. ANIPIO formally contacted 50 Specialist Nurses who specialize in infection control and risk assessment. These specialists were strategically selected to represent all 20 Italian regions, ensuring a geographically diverse sample. Each specialist nurse was then tasked with recruiting additional participants within their respective regions, creating a snowball sampling effect. This method aimed to achieve a broad and representative sample of clinical nurses across Italy, leveraging the professional networks of infection control specialists to reach a diverse array of nursing professionals.

Inclusion criteria for participants were: (i) qualification as a registered nurse (RN); (ii) active involvement in clinical practice, and (iii) consent to participate in the study. No minimal post-registration clinical experience requirement was considered. Each Specialist Nurse was required to enrol the medical, geriatric, rehabilitation, general surgical, orthopaedic, and intensive care units, and to indicate how many potential RNs would have completed the questionnaire. No minimal post-registration clinical experience requirement was set.

### Questionnaire

The coordinating centre of the research network developed the tool by considering a recent literature review [12]. It included the following sections:

I) Individual variables of participants (e.g., age, gender, level of education);
II) 14 questions about the knowledge of RNs regarding antimicrobial resistance and stewardship (e.g., *Can an infection from MDRO (Multi-Drug Resistant Organisms) prolong hospital stays and increase costs?*);
III) eight questions related to the attitude of RNs towards AMS activities and programs (e.g., *Is contributing to the selection of the most appropriate route of antibiotic administration for the patient part of the nurse’s competencies?*);
IV) seven questions about RNs practices (e.g., *Do you educate the patient/caregiver on the appropriate management of the antibiotic(s)?*);
V) ten questions about structural variables (e.g., *Is there a computerized alert system that monitors clinical data related to potential infections?*); and.
VII) three questions about process that promote AMS implementation (e.g., *Do group dynamics influence the implementation of antimicrobial stewardship?*).

The tool is based on 42 questions with a five-option frequency (i.e., Likert 1–5, with 1 representing “Strongly Disagree,” 2 representing “Disagree,” 3 representing “Neutral” or “Neither Agree nor Disagree,” 4 representing “Agree,” and 5 representing “Strongly Agree”) and dichotomous (i.e., yes/no; true/false) scales. We categorized knowledge scores into adequate (Likert 3–5) and inadequate (Likert 1–2) groups, with 3–5 indicating satisfactory knowledge and 1–2 reflecting lower knowledge levels. Similarly, attitude scores were divided into positive (Likert 3–5) and negative (Likert 1–2) categories, representing favorable and less favorable attitudes, respectively.

A pilot study was conducted to assess the face and content validity, as well as the reliability, of the existing 42-item questionnaire. A group of 46 expert nurses participated in this evaluation process. Face and content validity [16] were qualitatively assessed for clarity, comprehensibility, and appropriateness of the survey instrument. Each item was examined and rated on its clarity, relevance, and importance. To establish test-retest reliability, the same 46 participants were invited to complete the questionnaire again after a two-week interval. The test-retest reliability coefficient for the questionnaire was 0.86, indicating good stability over time. Based on the pilot study results, some semantic adjustments were made, and overlapping questions were addressed. However, the core structure of the questionnaire remained intact, with no items being removed.

### Data collection

The questionnaire was created using Microsoft Forms^®^ and was distributed by the authors through the Specialist Nurses, outlining the objectives and ensuring participants about the anonymity of their responses. Additionally, measures were taken to guarantee that only the authors had access to the data. The form was made available on March 1, 2023, and remained open for three months until May 31. The authors also took responsibility for sending reminders on a weekly basis.

### Data analysis

The data analyses were conducted using SPSS version 27, IBM Corp., Armonk, NY, USA. Descriptive statistics was performed by calculating frequencies, percentages, averages (Confidence Interval [CI] 95%). Data were stratified according to the clinical setting (e.g., medical unit). Differences across groups were explored by using Chi Square and t-test or ANOVA, depending on the nature of the variables. Furthermore, differences at the participant level (e.g., age, gender, level of education) were examined. Pearson coefficient analysis was used to identify correlation between the knowledge, attitude, and practices scores, while multiple linear regression was used to examine the relation between the significant factors of the univariate analysis with AMS attitude, practice and structure. A significance threshold of *p* ≤.05 was applied to all analyses.

### Ethical issue

The research protocol was first developed and approved by the research group of the Italian Society for Professionals in Infection Control (see authors) and the national network of specialist nurses. Before starting the study, written permissions were obtained from the University of Parma’s Ethics Committee (REB - RESEARCH ETHICS BOARD, prot. n. 16847, 19.01.23).

The questionnaire was sent via e-mail by each Specialist Nurse of the Italian Society for Professionals in Infection Control to all nurses involved. Eligible participants were free to participate or not to the survey and no rewards will be offered. In the initial part of the questionnaire, there were ensured the information regarding the aims of the study, the confidentiality of the data collected and its anonymity also with regards to the health care facilities where participants were involved. Each participant was invited to express formally the consent or not to participate. Then, after having answered, the questionnaire was displayed and participants were allowed to fill in it.

### Sources of bias assessment

There were four potential sources of bias [15] in this study, and the authors took specific measures to minimize them. Firstly, to address the *coverage bias* the authors used the network of the Italian Society for Professionals in Infection Control to select samples that are more representative of the population of clinical RNs. Secondly, to overcome *sampling bias* the authors clearly defined the target population and adjusted the analysis and interpretation accordingly. Thirdly, to address the *non-response bias* the authors sent email reminders on a weekly basis. Lastly, to overcome the *measurement bias* the authors conducted a pilot test of the instrument.

## Results

The Specialist Nurses successfully identified a potential pool of 3,250 clinical nurses across Italy. From this pool, 1,651 nurses participated in the survey, resulting in a response rate of 50.8%. The demographic characteristics of the participants are outlined in Table 1. The sample comprised 1,265 females (77.6%) and 375 males (22.7%). In terms of educational attainment, 73.8% of the registered nurses (RNs) held basic education (e.g., bachelor’s degree), while 26.2% had advanced education (e.g., master’s degree or higher). The average professional experience of the participants was 15 years. They were employed across various medical specialties, including medical (37.0%), intensive care (27.0%), orthopaedics (14.0%), surgery (13.5%), geriatric (6.0%), and rehabilitation (2.0%). The participating nurses represented 18 out of the 20 Italian regions. Referral hospitals were categorized by size into three types: first-level hospital (34.3%), second-level hospital (37.1%), and basic hospital (28.6%).

**Table 1:** Survey demographics (*n* = 1,651)

Variable	
Age, mean (SD)	39.4 (10.9)
Gender, *n*. (%)	
Female	1265 (76.6)
Male	375 (22.7)
Non-binary	11 (0.7)
Education, *n*. (%)	
Bachelor’s degree in nursing	869 (52.6)
Nursing diploma	350 (21.2)
One-year post-bachelor course	305 (18.5)
Master’s degree in nursing	103 (6.2)
One-year post-master course	18 (1.1)
PhD	6 (0.4)
Working experience (years), mean (SD)	15.1 (12.4)
Workplace, *n*. (%)	
Medical	606 (36.7)
Intensive care	445 (27.0)
Orthopedic	236 (14.3)
Surgical	223 (13.5)
Geriatric	106 (6.4)
Rehabilitation	35 (2.1)
Italian region, *n*. (%)	
Emilia-Romagna	268 (16.2)
Lombardia	221 (13.4)
Lazio	166 (10.1)
Toscana	158 (9.6)
Sicilia	155 (9.4)
Marche	136 (8.2)
Veneto	128 (7.8)
Trentino-Alto Adige	107 (6.5)
Abruzzo	87 (5.3)
Friuli-Venezia Giulia	71 (4.3)
Puglia	54 (3.3)
Liguria	44 (2.7)
Umbria	34 (2.1)
Campania	14 (0.8)
Calabria	3 (0.2)
Piemonte	3 (0.2)
Basilicata	1 (0.1)
Sardegna	1 (0.1)
Hospital size, *n*. (%)	
Second level Hospital: 300,000–1,200,000	611 (37.1)
First level Hospital: 150,000-300,000	567 (34.3)
Basic Hospital: 80,000-150,000	473 (28.6)

SD, Standard Deviation; PhD, Philosophiae Doctor

Table 2 displays scores related to AMS knowledge, attitude, and practices, categorized by demographic variables and workplace characteristics. Notably, factors such as gender, age, education level, and the size of the hospital where nurses are employed did not exhibit statistical significance concerning their knowledge, attitudes, and practices in AMS. However, the workplace, particularly in the surgical area, showed statistical significance regarding practices (*P* =.001). Additionally, the region of origin (north-central) showed significance concerning attitudes (*P* =.001). No statistically significant differences were observed between structural variables and AMS process variables based on workplace characteristics (refer to Table 3).

**Table 2:** AMS knowledge, attitude and practice scores by demographic variables and workplace characteristics

Variable	Knowledge			Attitude			Practices		
	Adequate	Not adequate	*p*-value	Positive	Negative	*p*-value	Yes	No	*p*-value
Gender, *n*. (%)									
Male	367 (97.9)	8 (2.1)	0.723	273 (72.8)	102 (27.2)	0.992	337 (89.9)	38 (10.1)	0.198
Female	1231 (97.3)	34 (2.7)		925 (73.1)	340 (26.9)		1105 (87.4)	160 (12.6)	
Non-binary	11 (100.0)	0 (-)		8 (72.7)	3 (27.3)		11 (100.0)	0 (-)	
Age, mean (SD)	39.3 (10.9)	40.6 (10.4)	0.458	39.4 (10.8)	39.2 (11.0)	0.715	39.4 (11.0)	39.1 (10.0)	0.685
Education ^a^, *n*. (%)									
Basic	1187 (97.4)	32 (2.6)	0.725	902 (74.0)	317 (26.0)	0.145	1088 (89.3)	131 (10.7)	0.009
Advanced	422 (97.7)	10 (2.3)		304 (70.4)	128 (29.6)		365 (84.9)	67 (15.5)	
Workplace ^b^, *n*. (%)									
Medical	734 (98.3)	13 (1.7)	0.146	552 (73.9)	195 (26.1)	0.445	662 (88.6)	85 (11.4)	< 0.001
Surgical	443 (96.5)	16 (3.5)		325 (70.8)	134 (29.2)		426 (92.8)	33 (7.2)	
Intensive care	432 (97.1)	13 (2.9)		329 (73.9)	116 (26.1)		365 (82.0)	80 (18.0)	
Hospital ^c^, *n*. (%)									
Second level	598 (97.9)	13 (2.1)	0.711	432 (70.7)	179 (29.3)	0.117	524 (85.8)	87 (14.2)	0.068
First level	551 (97.2)	16 (2.8)		413 (72.8)	154 (27.2)		511 (90.1)	56 (9.9)	
Basic	460 (97.3)	13 (2.7)		361 (76.3)	112 (23.7)		418 (88.4)	55 (11.6)	
Region ^d^, *n*. (%)									
Central-Northern Italy	978 (97.8)	22 (2.2)	0.271	773 (77.3)	227 (22.7)	< 0.001	875 (87.5)	125 (12.5)	0.432
Central-Southern Italy and Islands	631 (96.9)	20 (3.1)		433 (66.5)	218 (33.5)		578 (88.8)	73 (11.0)	

AMS, Antimicrobial Stewardship; SD, Standard Deviation

^a^ Basic: Bachelor’s degree in nursing, Nursing diploma. Advanced: One-year post-bachelor course, Master’s degree in nursing, One-year post-master course, PhD

^b^ Medical area includes: Medical area, Geriatric and Rehabilitation area. Surgical area includes: Orthopedic and General Surgery area

^c^ Second level Hospital: 300,000–1,200,000. First level Hospital: 150,000–300,000. Basic Hospital: 80,000–150,000. Hospital classification from www.gazzettaufficiale.it/eli/id/2015/06/04/15G00084/sg

^d^ Central-Northern Italy: Lombardia, Friuli Venezia-Giulia, Veneto, Trentino-Alto Adige, Emilia-Romagna, Piemonte, Liguria and Toscana. Central-Southern Italy and Islands: Lazio, Marche, Puglia, Sicilia, Abruzzo, Calabria, Umbria, Campania, Basilicata, Sardegna

**Table 3:** AMS structure and process scores by workplace characteristics

Variable	Structure			Process		
	Yes	No	*p*-value	Yes	No	*p*-value
Workplace ^a^, *n*. (%)						
Medical	388 (51.9)	359 (48.1)	0.015	613 (82.1)	134 (17.9)	0.589
Surgical	209 (45.5)	250 (54.5)		387 (84.3)	72 (15.7)	
Intensive care	244 (54.8)	201 (45.2)		367 (82.5)	78 (17.5)	
Hospital ^b^, *n*. (%)						
Second level	289 (47.3)	322 (52.7)	0.052	504 (82.5)	107 (17.5)	0.081
First level	308 (54.3)	259 (45.7)		484 (85.4)	83 (14.6)	
Basic	244 (51.6)	229 (48.4)		379 (80.1)	97 (19.9)	
Region ^c^, *n*. (%)						
Central-Northern Italy	531 (53.1)	469 (46.9)	0.029	822 (82.2)	178 (17.8)	0.425
Central-Southern Italy and Islands	310 (47.6)	341 (52.4)		545 (83.7)	106 (16.3)	

AMS, Antimicrobial Stewardship; SD, Standard Deviation

^a^ Medical area includes: Medical area, Geriatric and Rehabilitation area. Surgical area includes: Orthopedic and General Surgery area

^b^ Second level Hospital: 300,000–1,200,000. First level Hospital: 150,000–300,000. Basic Hospital: 80,000–150,000. Hospital classification from www.gazzettaufficiale.it/eli/id/2015/06/04/15G00084/sg

^c^ Central-Northern Italy: Lombardia, Friuli Venezia-Giulia, Veneto, Trentino-Alto Adige, Emilia-Romagna, Piemonte, Liguria and Toscana. Central-Southern Italy and Islands: Lazio, Marche, Puglia, Sicilia, Abruzzo, Calabria, Umbria, Campania, Basilicata, Sardegna

Pearson correlation was conducted to examine the relationship between the knowledge, attitude, and practice scores of the respondents (see Table 4). The analysis revealed that knowledge was more positively related to attitude (*r* =.339; *P* <.001) than to practices (*r* =.072; *P* <.003). Conversely, attitude showed a positive correlation with practices (*r* =.105; *P* <.001). A multiple linear regression was performed to predict the total attitude toward AMS based on Italian geographical areas (Table 5), and the results indicated that the model was not statistically significant (F = 0.018, *P* >.05). Regarding practices, the results of the regression indicated that the model was significant, as predicted by the education level and workplace (F = 24.9, *P* <.001). Nurses working in surgical areas scored higher than those working in medical wards (β = 0.467, *P* <.01), while critical care nurses scored lower (β= -0.398, *P* <.01). The results showed that structure scores as predicted by workplace, hospital and region were significantly affected (F = 4.64, *P* <.001). In particular, surgical nurses scored lower than their medical counterparts (β= -0.315, *P* <.01).

**Table 4:** Pearson correlation between knowledge, attitude, and practices scores

		Knowledge	Attitude	Practices
Knowledge	r Pearson	1		
	*p*-value	-		
Attitude	r Pearson	0.339**	1	
	*p*-value	< 0.001	-	
Practices	r Pearson	0.072*	0.105**	1
	*p*-value	0.003	< 0.001	-
	*n*.	1651	1651	1651

* *p* <.01, ** *p* <.001

**Table 5:** Results of multiple linear regression on significant factors associated with AMS attitude, practice and structure

Variable	Coefficient (β)	Standard error	F	*p*-value	95.0% Confidence Interval	
					Lower bound	Upper bound
**Attitude**			0.018	0.893		
Region (Central-Southern Italy and Islands)	-0.003	0.023			-0.049	0.043
**Practice**			24.9	< 0.001		
Educational level (postgraduate)	-0.152	0.088			-0.326	0.020
Workplace (surgical)	0.467*	0.092			0.284	0.648
Workplace (intensive care)	-0.398*	0.094			-0.583	-0.213
**Structure**			4.64	< 0.001		
Workplace (surgical)	-0.315*	0.145			-0.601	-0.030
Workplace (intensive care)	0.156	0.148			-0.135	0.447
Hospital (first level)	0.291	0.155			-0.012	0.596
Hospital (second level)	-0.265	0.154			-0.567	0.037
Region (Central-Southern Italy and Islands)	-0.064	0.126			-0.311	0.181

* *p* <.01

## Discussion

To the best of our knowledge, this is the first large-scale, nationwide study in Italy to quantitatively report on nurses’ knowledge, attitudes, practices, and perceived structure and process scores specifically related to AMS. While previous research has often focused on the healthcare workforce as a whole [6], or included a significant proportion of students [13], our study addresses the gap in literature specifically targeting clinical nurses [7]. Moreover, it explores factors that may promote or hinder nurse involvement in AMS activities, an area where little was previously known. Unlike earlier studies that revealed limited familiarity with AMS among nurses [8, 9], or focused on specific regions [11], our research provides a comprehensive, country-wide perspective on Italian nurses’ competencies in AMS. By examining both internal factors (knowledge, attitudes, practices) and external factors (structures and processes) [12], this study offers a multifaceted view of nursing proficiency in AMS within the Italian healthcare context. Furthermore, our study achieved a favorable response rate of 50.8%, which is noteworthy when compared to other similar studies reporting lower response rates [8]. The demographic composition of the sample revealed a predominantly female cohort (77.6%), with an average work experience of 15 years. Regarding education, our findings are consistent with patterns observed in similar studies [8, 17].

Statistical analyses were performed to identify associations between demographic and workplace variables and AMS knowledge, attitudes, and practices. Notably, gender, age, education level, and hospital size did not yield statistically significant differences. Nevertheless, workplace characteristics and regional distinctions were observed to impact of some dimensions of AMS. Firstly, when analyzing the workplace, we categorized the sample into medical, surgical, and intensive care settings. Surgical settings exhibited a higher inclination toward AMS, while intensive care exhibited a lower inclination, compared to medical settings. Additionally, surgical settings showed less organizational structure than medical settings.

The intensive care unit poses unique challenges related to antibiotics due to the distinct characteristics of its patient population and setting. These challenges include clinical complexity and case mix, pharmacokinetic variability, environmental factors (e.g., different pathogens), and the need for individualized management of patients that goes beyond general guidelines [18]. Given these complexities, it is reasonable to anticipate lower adherence to AMS practices in this setting.

Unsurprisingly, surgical nurses reported more proficient practices in AMS. Given the prevalent occurrence of surgical site infections, accounting for 38% of nosocomial infections [19], healthcare professionals in this setting prioritize optimizing surgical prophylaxis and antimicrobial therapy as their primary objective. However, surgical settings appeared to face challenges due to a lack of hospital support in tracking usage on an ongoing basis through necessary infrastructure, such as the formation of a surgical antibiotic stewardship interdisciplinary team [20]. Furthermore, regional disparities, specifically between central-northern and south-northern Italy, were observed. The northern regions showed a greater structural availability, although this discrepancy wasn’t conclusively supported in the multiple linear regression analysis. This discrepancy may highlight a gap in the implementation of AMS. It’s important to note that our study faced challenges in achieving uniform representation across all Italian regions, with two regions lacking representation and an uneven distribution of participants between central-northern and south-northern Italy. These geographical variations in participation and AMS readiness underscore the complexity of implementing nationwide AMS strategies and the need for targeted, region-specific approaches in enhancing antimicrobial stewardship in nursing practice across Italy.

In other countries, factors such as underfunded public health, hospital infrastructure, behavioral determinants, and contextual factors have been previously documented, indicating variations in the developmental stage of AMS across different regions [21]. In the Italian context, additional factors contribute to this diversity. The normative autonomy granted to regions in healthcare governance leads to varied AMS implementation priorities [22]. The presence of private healthcare facilities alongside public ones, each with distinct funding models, further complicates the landscape. Moreover, diverse accountability measures and quality payment systems among regions influence the emphasis placed on AMS initiatives. These factors, unique to Italy’s healthcare system, add layers of complexity to the implementation of AMS strategies and explain, in part, the regional disparities observed in our study. The differences we observed highlight the need for customized interventions and educational programs in each healthcare setting. Existing research supports the idea that a deeper understanding of the context improves the quality of research and knowledge assimilation. This, in turn, facilitates the translation of evidence-based health interventions into everyday clinical practice [23]. Our findings confirmed the importance of tailoring AMS interventions based on specific contexts rather than adopting one-size-fits-all national programs.

Additional analysis incorporated Pearson correlation. However, a correlation of 0.339 is deemed weak, while the correlations of 0.072 and 0.105 are considered not meaningful. These results underscore possible intricate relationships between the cognitive aspects of knowledge and attitudes and the practical implementation of AMS principles. Notably, these findings deviate slightly from the literature, which typically places greater emphasis on the foundational role of knowledge [17]. Overall, we have recorded knowledge scores consistently exceeding 90% (refer to the “Adequate Knowledge” column in Table 2). Examining attitudes, the scores consistently register at 70% (refer to the “Positive Attitude” column in Table 2). Therefore, the question arises whether, at this stage, the knowledge is sufficient, or if there is a need to address practical behaviors and the facilitating factors.

Current trends assert that the behavioral science approach and qualitative methods synergize effectively, making valuable contributions to AMS/AMR research [24]. Following Borek et al. [25], recommendations for enhancing the use of the behavioral science approach in this field include: understand behaviors and determinants before intervention; utilize diverse qualitative methods for comprehensive insights (e.g., focus groups, interviews, workshops); consistently apply behavior change theories and frameworks in interventions; involve stakeholders and target populations in all stages; incorporate qualitative and mixed-methods process evaluations in AMS intervention studies. Human behaviors, encompassing attitudes and actions, significantly influence both the propagation and mitigation of AMR, perhaps more so than knowledge alone. Additionally, concerning external factors, we can hypothesize that cultural, organizational, and technical elements within the clinical setting may influence nurses’ behavior regarding AMS. Addressing these factors is crucial to enhance best practices and achieve positive outcomes for patients.

### Limitations

This study is subject to certain limitations. Firstly, it is crucial to emphasize that one limitation lies not in the quantitative aspect itself but rather in the qualitative composition of the sample, as evidenced by a non-response rate of approximately 50%. This suggests potential variations in engagement or indifference among certain nurses regarding the subject matter. This could potentially have led to lower scores in attitudes, knowledge, and practices. Therefore, we can hypothesize that those who responded had greater knowledge and attitudes. To enhance survey response rates, researchers might contemplate reducing the number of survey items [26]. Then, since cross-sectional studies capture data at a single time point, establishing causal relationships between variables becomes difficult. Slightly correlations have been observed, but causation cannot be definitively determined. In addition, the potential limitation of our study also lies in the restricted generalizability of results due to observed regional differences within the country. Additionally, variations in population characteristics, healthcare systems, and cultural factors between countries may further impact the external validity of our findings. Lastly, a significant limitation lies in the instrument’s design, which was developed by the researchers. Instead of utilizing an existing instrument, we opted to create a new one to gather more specific information tailored to the context of our country. Furthermore, formal statistical methods for validating the tool, such as Cronbach’s alpha, have not been conducted.

### Implications for practice

Healthcare organizations must prioritize educational initiatives tailored to specific clinical settings and regional contexts, addressing both internal factors like knowledge and attitudes, as well as external factors related to structures and processes. Empowering nurses through comprehensive training programs, fostering interdisciplinary collaboration, and implementing supportive infrastructure can significantly contribute to responsible antimicrobial use and mitigate the threat of antimicrobial resistance. Nurses, as frontline caregivers, have a unique opportunity to drive positive change by actively participating in antimicrobial stewardship efforts, promoting patient education, and advocating for evidence-based practices within their respective healthcare facilities.

## Conclusions

In conclusion, this study enhances our understanding of the complex factors influencing nurses’ knowledge, attitudes, and practices in AMS. The significance attributed to workplace and regional variations underscores the necessity for targeted interventions and educational programs aimed at enhancing AMS competencies among healthcare professionals. Additionally, it is crucial to focus on attitude as an internal focal point, as it directly influences nurses’ behaviors and practices. Such initiatives have the potential to yield positive implications for improving patient outcomes and mitigating the emergence of antimicrobial resistance.

## Data Availability

No datasets were generated or analysed during the current study.
